# Reproductive Traits and Hatchling Characteristics of the Endemic Sardinian Grass Snake (*Natrix helvetica cetti*): First Field Data, with Screening for *Ophidiomyces ophidiicola*

**DOI:** 10.3390/ani15030418

**Published:** 2025-02-03

**Authors:** Matteo Riccardo Di Nicola, Luca Colla, Sergio Mezzadri, Anna Cerullo, Giuseppe Esposito, Paolo Pastorino, Giovanni Paolino, Pierluigi Acutis, Daniele Marini, Francesco Paolo Faraone

**Affiliations:** 1 Istituto Zooprofilattico Sperimentale del Piemonte, Liguria e Valle d’Aosta, Via Bologna 148, 10154 Turin, Italy; giuseppe.esposito@izsplv.it (G.E.); paolo.pastorino@izsplv.it (P.P.); pierluigi.acutis@izsplv.it (P.A.); 2 Wildlife Health Ghent, Faculty of Veterinary Medicine, Ghent University, Salisburylaan 133, 9820 Merelbeke, Belgium; 3 Department of Chemistry, Life Sciences and Environmental Sustainability, University of Parma, Parco Area delle Scienze, 43124 Parma, Italy; luca.colla1@studenti.unipr.it; 4 Via Palmerio, 29121 Piacenza, Italy; sergio.mezzadri@libero.it; 5 Department of Veterinary Sciences, University of Turin, Largo Paolo Braccini 2, 10095 Grugliasco, Italy; 6 Unit of Dermatology and Cosmetology, IRCCS San Raffaele Hospital, Via Olgettina 60, 20132 Milan, Italy; paolino.giovanni@hsr.it; 7 Department of Veterinary Medicine, University of Perugia, Via San Costanzo 4, 06126 Perugia, Italy; daniele.marini@dottorandi.unipg.it; 8 Department of Organismal Biology, Evolutionary Biology Centre, Uppsala University, Norbyvägen 18A, 752 36 Uppsala, Sweden; 9 Department of Biological, Chemical and Pharmaceutical Sciences and Technologies, University of Palermo, Via Archirafi, 18, 90123 Palermo, Italy; francescopaolo.faraone@unipa.it

**Keywords:** biometrics, body size, offspring, snake fungal disease, ophidiomycosis, Oo, Mediterranean, Natricidae, Sardinia, Italy

## Abstract

The Sardinian grass snake, *Natrix helvetica cetti*, is an island-endemic subspecies with a restricted and highly fragmented distribution confined to Sardinia, Italy. Information on its reproductive biology and wild offspring remain scarce in the scientific literature. This present study reports the first recorded data on a clutch of eggs laid by a wild melanistic female *N. h. cetti* that exhibited lethargy when observed basking, prompting a brief period of health monitoring and screening for the presence of the pathogenic fungus *Ophidiomyces ophidiicola* (Oo). The clutch yielded nine hatchlings, for which phenotypic data are provided and compared with existing information on the *Natrix natrix* complex. Both the adult female and its offspring tested negative in the Oo screening. The body size of the adult grass snake, specifically its snout-to-vent length of 48.3 cm, is the smallest ever recorded for a gravid female *N. helvetica*. This may indicate that maturity is reached at particularly small sizes, a distinctive trait of this intriguing island subspecies, suggesting the need for further investigation.

## Simple Summary

The Sardinian grass snake (*Natrix helvetica cetti*) is an endangered subspecies endemic to Sardinia, Italy, with a highly fragmented distribution. This study reports the first documented clutch from a wild melanistic female, yielding nine healthy hatchlings. Detailed phenotypic and biometric data were collected and compared to other *Natrix natrix* complex species, revealing the smallest recorded snout-to-vent length for a gravid female *N. helvetica*, suggesting unique traits for this subspecies. Both the dam and hatchlings tested negative for *Ophidiomyces ophidiicola*, the pathogen responsible for ophidiomycosis. These findings address significant gaps in knowledge about the reproductive biology of *N. h. cetti*, offering helpful insights for the conservation and management of this rare and vulnerable subspecies.

## 1. Introduction

The reptiles of the Sardinian–Corsican island system display a composite biogeography, comprising species likely introduced by human activity [1,2,3,4] and endemic taxa [5,6,7,8]. The Sardinian grass snake, *Natrix helvetica cetti*, is an endemic snake subspecies with a highly fragmented and restricted geographic distribution, primarily in the highlands of the southern and eastern part of Sardinia main island, Italy [9,10,11]. It has been classified as ‘endangered’ in the latest assessment by the Italian Committee for the IUCN [12]. For a summary of the current taxonomic status of the subspecies, see Section 3.1.1. in Di Nicola et al. and references therein [11]. Research on *N. h. cetti* is scarce, particularly in comparison to that of other members of the *Natrix natrix* complex, and a huge knowledge gap regarding its reproductive biology has been highlighted [10,11]. It is known that it is an oviparous snake, with a reproductive cycle analogous to that of the mainland *Natrix natrix* complex [10], and, at birth, *N. h. cetti* hatchlings measure around 15 cm in length [13]. Regarding the *Natrix natrix* complex reproduction, it is known that mating begins in early spring (see Table 1), with frequent occurrences of mating balls [14,15,16]. The oviposition occurs from June to September, with clutch sizes ranging from four to one-hundred-and-five and an average of nine; the average egg dimensions are 9–24 × 21–40 mm, with a weight of 3–5 g [15,16,17,18,19,20,21]. Collective oviposition of up to 4000 eggs are known [15,22]. Eggs are laid under the soil, under decomposing wood or inside manure piles [16,18,19,23]. In some cases, females also use artificial objects, such as plastic sheets, to deposit their clutches [24]. The incubation period ranges from 22 to 77 days depending on the temperature [16,19,20,21,22,23,25]. On average, hatchlings have a length of 11–25 cm and weigh 2.5–5 g, depending on the incubation temperature [15,18,19,21,23,25]. Idrisova and Khairutdinov [25] identified excessively high incubation temperature as a significant factor influencing deviations in pholidosis, occurrence of malformations and variations in hatchling colouration.

In the present study, we provide the first detailed description of an oviposition event in *N. h. cetti*, including biometric data of the gravid female and its clutch, comparing these findings to existing data on the *N. natrix* complex. In this species complex, reproductive success for both males and females appears to be positively correlated with body size, and clutch size is positively correlated with female size (see [14,15,16,17,18,19,20,21]). However, in island populations, a reduction in female size is often observed [26,27,28]; hence, we expect a similar decrease in size at maturity for *N. h. cetti*.

The adult female was observed during a screening for the presence of *Ophidiomyces ophidiicola* (Oo), the pathogenic fungus responsible for ophidiomycosis (see [29,30,31]), which has not yet been detected in Sardinia ([32]; Di Nicola et al., in preparation). As she exhibited apparently lethargic behaviour, she was temporarily held for a clinical examination, and both the dam and the hatchlings were screened for the presence of Oo.

**Table 1:** Literature data on reproduction in the *Natrix natrix* complex. Hatchling size is expressed in total length, except where indicated otherwise in brackets. *N. n.* = *N. natrix*; *N. a.* = *N. astreptophora*; *N. h.* = *N. helvetica*.

Taxon	Oviposition Period	Clutch Size	Egg Dimensions (mm; g)	Incubation	Hatchling Size (cm; g)	Reference
*N. n.* complex		6–105	23–40 × 13–20	30–75 days	12–22; 3	[18]
*N. n.* complex	Jun–Jul	6–70	20–40 × 9–24	3–11 weeks	11–22	[19]
*N. n.* complex	Jun–Aug	8–32	21–40 × 11–24; 3–5	30–33 days		[15]
*N. n.* complex	Early summer	12–50		45–50 days		[20]
*N. n.* complex		8–40			15–18	[33]
*N. a.*	Late Jun–Early Jul	6–50		60 days	14.5–21.6	[34]
*N. a.*		9–26				[35]
*N. a.*	Late Jun–Early Jul	12–29			15.2 (SVL)	[36]
*N. n. vulgaris*	Late Jul	4–24		22–45 days	19–22; 2.5–5	[21]
*N. n. scutata*	Jun–Jul	4–13	43 × 13; 5.8	10 weeks		[16]
*N. n. scutata*				25–52	16–25; 2–5	[25]
*N. h. helvetica*	Jun–Sep	11–53	21–37 × 11–24	3–8 weeks	15–21	[17]
*N. h. helvetica*				6–10 weeks		[37]
*N. h. helvetica*		13–47			16.6–19	[38]
*N. h. helvetica*	Late Jun–Early Jul	8–40				[39]
*N. h. helvetica*		70				[40]
*N. h. helvetica*		28		31–63 days		[41]
*N. h. cetti*	Early Jul	9	27–44 × 16–19	44 days	18.5–21.1; 2.7–3.8	*This study*

## 2. Materials and Methods

### 2.1. Field Sampling and Phenotypic Processing

**Figure 1:**
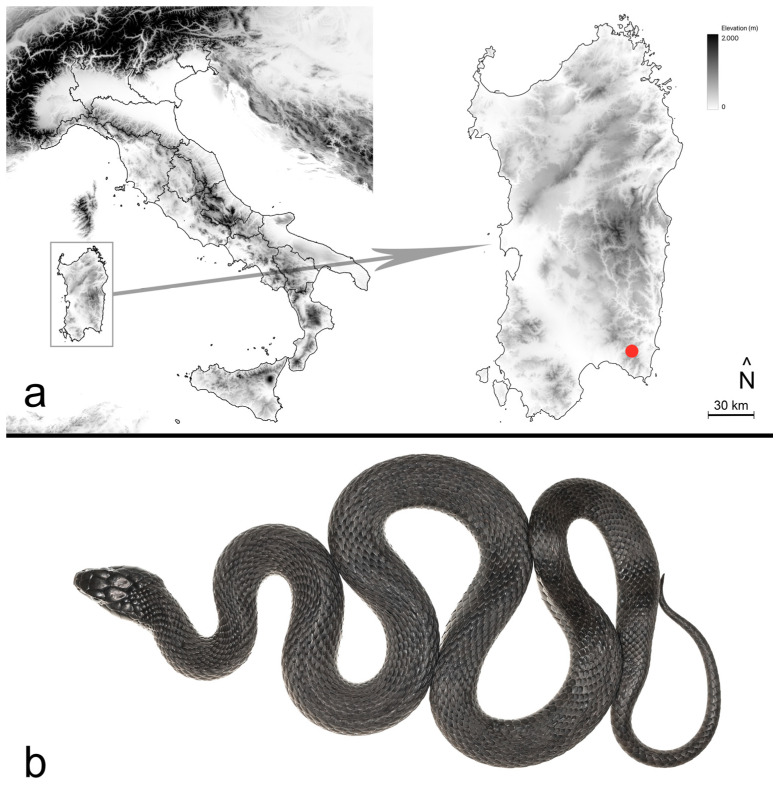
Map of Italy divided into regions, with a detailed inset of Sardinia on the right. The red dot indicates the area of Sette Fratelli, Sardinia, where the gravid female *Natrix helvetica cetti* was found (**a**). Dorsal view of the melanistic adult female after egg-laying (**b**).

The fieldwork, which included the skin swabbing of potentially captured snakes, was carried out to investigate the presence of the pathogen *O. ophidiicola* among free-ranging snakes in southwestern Sardinia. This survey took place within the Sette Fratelli forest complex (approximate coordinates: 39°18′ N, 9°24′ E, WGS 84; Figure 1a) following a dirt path through a *Quercus ilex* L. forest on a northwest-facing slope. The weather was clear, with moderate wind (averaging around 21 km/h) and temperatures ranging from a minimum of 19 °C to a maximum of 28 °C.

At approximately 10:30 am, a melanistic adult female *N. h. cetti* was found along a path’s edge near shelters formed by shrubs and rocks (altitude: about 560 m a.s.l.), basking almost fully exposed to the sun. Despite being warm from sun exposure, the snake displayed neither escape nor death-feigning behaviour when approached. At first glance, it appeared healthy, with no visible skin lesions and a slightly enlarged terminal trunk segment, consistent with a gravid individual. However, due to its unusually lethargic behaviour, a decision was made to temporarily detain the snake for further veterinary monitoring, in accordance with the health screening procedures established in our fieldwork authorisation. The snake was provisionally housed in an 80 × 40 × 40 cm glass terrarium that had been thoroughly cleaned and disinfected prior to use. The set up included an absorbent paper substrate, a humid chamber filled with moss and wood fragments from the capture site, an artificial shelter and a water dish. Additionally, a timer-controlled UVB lamp was installed to mimic the seasonal light cycle (15 h of light).

The snake was tested for Oo presence using a double cutaneous dry swab. Every swab was performed with a single sterile cotton-tipped applicator with ten repetitions on the dorsal scales, ventral scales and head region to cover the whole skin surface. Dry swabs tips were placed in 1.5 mL tubs and subsequently stored at −20 °C. No invasive collection of skin fragments was carried out as the animal had no suspect skin lesions.

On 2 July 2023, four days after being detained, the snake laid a clutch of nine eggs that were promptly placed in an open container with moss as a substrate and maintained at a controlled temperature (range 26–28 °C) in an incubator. Following oviposition, the dam was kept under observation for two days (Figure 1b) and, after confirming its health status, was released at the exact location where it was found six days earlier. The release occurred mid-morning under favourable climatic conditions to ensure the snake could meet its thermal and light requirements (see [42,43]).

Given the documented cases of postnatal transmission of Oo from dams to offspring [44], every hatchling was tested for *Ophidiomyces* presence using only their postnatal ecdysis to minimise invasiveness via cutaneous dry swab or scale clipping. All hatchlings were kept under observation until completing their postnatal ecdysis. Following an accurate clinical examination of each individual, the hatchlings were released at the same location where the mother was captured. The release occurred one day after hatching, during mid-morning, to take advantage of favourable climatic conditions and ensure adequate exposure to heat and light [42,43]. Handling of the hatchlings was minimised by obtaining measurements from detailed macro photographs analysed using the software ImageJ (version 1.54k) [45]. Each snake was sexed by analysing the shape of the cloacal region and on the basis of the scale counts [10], and the following continuous and discrete measurements were taken (see [46,47]): snout–vent length (SVL, from the tip of the rostral scale to the cloaca); tail length (TL, from the cloaca to the tip of the tail); snout length (SL, from the tip of the rostral scale to the posterior end of the frontal scale); distance between nostrils (DBN); horizontal eye diameter (ED); pileus length (PL, from the tip of the rostral scale to the posterior margin of the parietal scales suture); and pileus width (PW, distance between the external margin of the parietal scales). Total length (TotL) was derived as the sum of SVL and TL. The body weight (BW) of all hatchlings was measured using a VEVOR HZ-B50002 balance (readability: 0.01 g). For the pholidotic analysis, the dorsal (DS), ventral (VS), subcaudal (ScS), supralabial (SS), preocular (PrS), postocular (PoS) and temporal scales (TS) were counted. Ventral scales were counted following the Dowling method [48]. The colour pattern of each snake was assessed following Di Nicola et al. [11]. To avoid any form of stress, individual eggs were measured only after hatching.

### 2.2. Statistical Analysis

Data normality was assessed using the Shapiro–Wilk test and diagnostic plots, and square root transformation was applied to reduce deviations from normality. A one-way ANOVA was performed to test for differences on biometric and meristic characters between sexes. Statistical analyses were conducted using the stats package and base R functions in R version 4.4.1 [49]; the box plots were created using Python 3.10 via Google Colab [50,51].

### 2.3. Laboratory Analysis

The presence of *Ophidiomyces ophidiicola* was evaluated using SYBR Green-based qPCR, following the methodology outlined by Marini et al. [32,52]. DNA was extracted from dry swabs (placed in 1.5 mL tubes at sampling) by adding 100 μL of PrepMan Ultra Sample Preparation Reagent (ThermoFisher, Carlsbad, CA, USA) and 50 mg of 0.5 mm zirconium oxide beads. To extract DNA from moults, 3 to 5 fractions (ca. 0.5 × 0.5 cm) were placed in 1.5 mL tubes by adding 50 μL of PrepMan Ultra Sample Preparation Reagent (ThermoFisher, Carlsbad, CA, USA) and 50 mg of 0.5 mm zirconium oxide beads. Samples were homogenised for 60 s using a Bullet Blender Storm 24 (Next Advance, Inc., New York, NY, USA) and then centrifuged at 13,000 RPM for 30 s; this process was repeated twice. The samples were subsequently heated at 95 °C for 13 min using a Techne^®^ Dri-Block^®^ DB-2D (Buch & Holm, Herlev, Denmark), cooled for 5 min and centrifuged again at 13,000 RPM for 30 s. Next, 50 μL of nuclease-free water was added, and after centrifugation under the same conditions, 50–75 μL of the supernatant was transferred to new 1.5 mL tubes. Following another round of centrifugation, 30–50 μL of DNA extract was obtained. DNA concentrations were measured Nanodrop 2000c spectrophotometer (ThermoFisher, Carlsbad, CA, USA). The DNA extracts were diluted to 12.5 ng/μL and used immediately. Adhering to the protocol from Marini et al. [32,52], each DNA sample was tested in triplicate using SYBR Green-based qPCR assays targeting the internal transcribed spacer 2 (ITS2) region of the ribosomal RNA gene complex (primers from Bohuski et al. [53]) and the mitochondrial NADH dehydrogenase subunit 1 (nad1) gene (primers from Lorch et al. [54]) specific to *O. ophidiicola*. Each 10 μL qPCR reaction comprised 5 μL of iQ SYBR Green Supermix (Bio-Rad Laboratories Inc., Hercules, CA, USA), 50 ng of DNA (4 μL of a 12.5 ng/μL solution), 0.7 μL of nuclease-free water and 0.3 μL of a 10 μM primer mix. Amplifications were conducted on a CFX385™ Touch Real-Time PCR Detection System (Bio-Rad Laboratories Inc., Hercules, CA, USA) with the following cycling conditions: initial denaturation at 95 °C for 3 min; 40 cycles consisting of 95 °C for 3 s and 60 °C for 30 s; followed by a melt curve analysis from 65 °C to 95 °C, increasing by 0.5 °C increments with readings every 5 s. The qPCR results, including Ct values, melting curves and relative fluorescence units (RFUs), were analysed using Bio-Rad CFX Maestro software 1.1 (v.4.1.2). Detailed information on PCR assay efficiency, the controls used and RFU analysis in the End-Point mode of the software is available in Marini et al. [32].

## 3. Results

**Figure 2:**
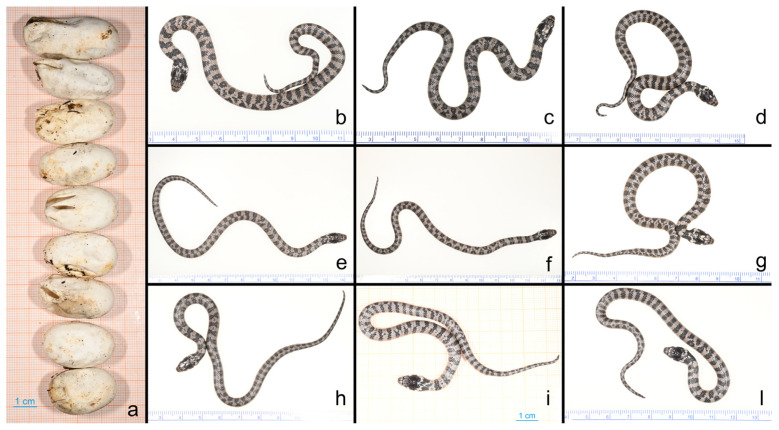
Eggs on graph paper after hatching (**a**) and the nine hatchling *Natrix helvetica cetti* only a few hours old (**b**–**l**).

**Figure 3:**
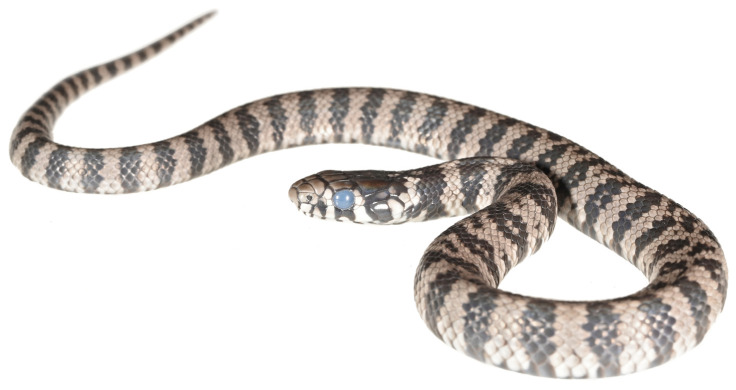
Hatchling in pre-ecdysis condition with dull-appearing skin and blue eyes.

The melanistic adult female Sardinian grass snake, which was found and detained for further observations, measured 58.8 cm in total length (48.3 cm SVL + 10.5 cm tail). Four days after being placed in captivity, the snake laid a clutch of nine eggs beneath moss litter. Following 44 days of incubation at 27–28 °C, the first egg hatched during the night, with the remaining eight eggs hatching within the subsequent 24–36 h, totalling nine hatchlings in apparently excellent health. The post-hatching egg lengths ranged from 2.7 to 4.4 cm (Figure 2). Six to eighteen hours post-hatching, all hatchlings displayed pre-ecdysis conditions, characterised by dull-appearing skin and blue eyes (Figure 3). The postnatal ecdysis process was completed within 24 h of hatching.

Five out of nine hatchlings were identified as females and four as males (Table 2).

**Figure 4:**
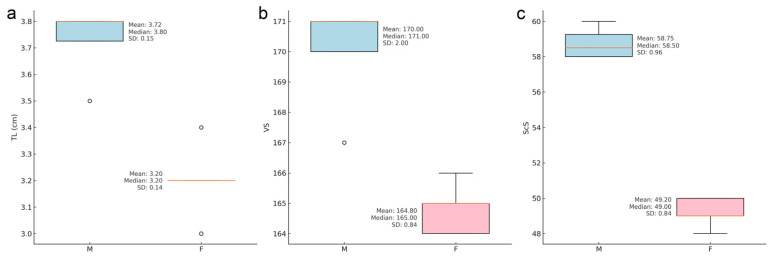
Differences in tail length (**a**), ventral scales (**b**) and subcaudal scales (**c**) between males and females in the nine *Natrix helvetica cetti* newborns.

The hatchlings measured between 18.5 to 21.1 cm in total length, with an average of 20.2 cm ± 0.8 cm standard deviation (SD). Males had significantly longer tails (TL) (mean ± SD: 3.7 ± 0.2 cm vs. 3.2 ± 0.1 cm; F = 28.51, *p* = 0.0010), more subcaudals (ScS) (58.8 ± 1.0 vs. 49.2 ± 0.8; F = 253.94, *p* = 0.0000) and more ventral scales (VS) (170.0 ± 2.0 vs. 164.8 ± 0.8; F = 28.50, *p* = 0.0010) compared to females (Figure 4). TL also determines a higher TotL in males (20.9 ± 0.19 cm vs. 19.6 ± 0.8 cm; F = 10.61, *p* = 0.0033), since there are no significant differences in SVL between the two sexes (16.9 ± 0.2 cm vs. 16.4 ± 0.8 cm; F = 1.73, *p* = 0.2262). No significant differences were found in the other analysed characters (Table 2).

Both the dam and the offspring tested negative for Oo molecular detection, as no specific Oo DNA was detected by qPCR, neither from the dry swabs of the adult female nor from the hatchlings’ shed skins.

## 4. Discussion

The Sardinian endemic grass snake subspecies, *Natrix helvetica cetti*, possesses numerous characteristics, including aspects of its reproductive biology, which remain largely unstudied. In this present study, we report data on a clutch of nine eggs laid by a melanistic adult female measuring 48.3 cm SVL. Regarding body size, this represents the smallest value recorded for a mature female of *N. helvetica*, as the literature reports minimum SLV values between 53 and 66 cm for this species [37,38,55]. For the other taxa of the *N. natrix* complex, minimum values greater than 60 cm SVL are usually reported for mature females [16,21,36,56]. However, in some Iranian populations of *N. natrix*, values as low as 35 cm SVL can be inferred, as noted by Ahmadzadeh et al. [57]. While our single data point is insufficient to establish a trend, it warrants further targeted investigations, as it suggests additional distinctive traits in this subspecies, which is already known for its smaller size compared to mainland populations [10,26,58,59]. Body size at maturity is an extremely plastic trait in snakes, especially in island populations, which can significantly diverge compared to mainland populations [60,61], sometimes in a very short time [62,63]. This was already observed for *N. natrix*, for which a decrease in size is also known in some island populations [27,28]. This dwarfism pattern and, sometimes, the reduction in sexual dimorphism, are possibly determined by the absence of large preys, such as the European toad, *Bufo bufo* (Linnaeus, 1758), which are usually selected by adult females [26,27]. In Sardinia, in fact, the presence of *B. bufo* was only recently confirmed due to local introduction events [64].

The clutch size, as well as the eggs’ dimensions, align with data from the *Natrix natrix* complex (Table 1). Despite oviposition occurring in a controlled environment, the female chose moss litter for egg-laying, corroborating preferences reported in earlier studies [16,18,21,23]. The incubation period lasted 44 days, which agrees with the range reported by previous authors (see Table 1) and closely matches the 42-day incubation period recorded by Townson [41] for *N. helvetica* under comparable conditions. The clutch size observed in this instance is lower than the range reported for the *N. natrix* complex (Table 1). This is consistent with the general trend in this species group, where the number of eggs is typically positively correlated with maternal size [16,21,38,56]. On the other hand, hatchlings’ body size in the *N. natrix* complex is usually considered not influenced by clutch size and maternal body size [21,56]. In line with this, the mean size of hatchlings of *N. h. cetti* both in terms of total length and weight (20.2 cm; 3.4 g) was comparable to values reported in previous studies for other species within the complex, even when incubation set-ups varied significantly [21]. Despite adult grass snakes being sexually dimorphic in size [28,56], hatchlings generally exhibit no differences in body size between sexes, also in terms of TotL [56]. Our findings confirmed this pattern in terms of SVL, as occurs in other populations of *N. helvetica* (cf. [55]), but not as regards TotL, influenced by the longer tail (TL) in males. This pattern is also observable in adults of the *N. natrix* complex, at least in the SVL/TL ratio [55,65,66,67]. The pholidotic characters reported here align with the range reported for this subspecies [10].

Wagner et al. [68] reported that for most Natricidae the postnatal ecdysis occurs immediately or within four days. Interestingly, all the *N. h. cetti* hatchlings featured in this study completed their postnatal ecdysis within a 24-h period of hatching. This aspect deserves further investigation, as it may have been influenced by the incubation parameters.

The melanistic colour pattern of the gravid female was compatible with what Goldenberg et al. [69] described as “charcoal” (see also [70]), and it was included in category IV by Di Nicola et al. [11]. Melanism, the most common form of colour polymorphism in snakes and animals in general [71], is typically associated in ectotherms with the thermal melanism hypothesis [69,72,73]. This hypothesis suggests that darker colouration enhances heat absorption, offering thermal advantages. However, the “charcoal” dark morphotype in *N. helvetica* is associated with high UV environments, implying a role in protection against solar radiation [69]. According to a recent review by Sahlean et al. [74], the ecological and functional traits of melanism in snakes have been studied by various authors, while the genetic bases are still not understood. Despite the melanistic colouration of the mother, none of the hatchlings in this study exhibited melanism. This may be because melanism usually does not develop at birth but emerges during ontogenesis (see [74,75]). Observations of *N. helvetica* populations in Sicily, where charcoal adults predominate, support that this morph is generally absent in yearlings (Faraone F.P., unpublished data).

However, the hatchlings exhibited a heavily barred pattern similar to category III as reported by Di Nicola and colleagues [11]. Additionally, none of the juveniles had a light-coloured collar.

The absence of *Ophidiomyces ophidiicola* in the monitored snakes aligns with other studies in Sardinia ([32]; Di Nicola et al. in preparation). While this finding is reassuring regarding the absence of the pathogen in this delicate insular ecosystem, limited research and reduced field efforts remain factors that may influence these results. Therefore, ongoing health surveillance for emerging infectious diseases (EIDs), including other fungal pathogens such as *Parananniziopsis* spp. [76], is essential.

Although this study sheds some light on *N. h. cetti* reproductive biology, it is crucial to emphasise the lack of data on this subspecies. To better understand and preserve this endemic snake subspecies, further research is urgently needed to study its biology, ecology and ethology. Such information is fundamental for an integrative approach to the study, monitoring and conservation of *N. h. cetti*.

## 5. Conclusions

The present work marks a pioneering exploration into the reproductive biology of the Sardinian grass snake, *Natrix helvetica cetti*. Our findings provide the first description of clutch characteristics and hatchling biometrics for this endangered subspecies, revealing notably small dam body size. Both the mother and her hatchlings tested negative for Oo, supporting that this pathogen does not currently pose a threat to the species in Sardinia. Future research should concentrate on longitudinal studies to track population trends and further explore the genetic and environmental factors affecting reproductive traits in this island-endemic subspecies. Additionally, it is important to continue health surveillance of EIDs to manage potential emergency conditions promptly.

## Figures and Tables

**Table 2:** Morphometric data for the nine *Natrix helvetica cetti* newborns, descriptive statistics and univariate results of the one-way ANOVA test between sexes. ^R^: right side; ^L^: left side; ^1^: first row; ^2^: second row, * = significant differences between sexes.

	juv. 1	juv. 2	juv 3.	juv. 4	juv. 5	juv. 6	juv. 7	juv. 8	juv. 9	Mean ± SD	*F*	*p*
Sex	M	F	M	F	M	F	M	F	F		df = 8	
TotL (cm) *	20.9	19.9	21.1	20.6	20.7	18.5	20.7	19.3	19.7	20.2 ± 0.8	9.5	0.0179
SVL (cm)	17.4	16.7	17.3	17.4	16.9	15.5	16.9	15.9	16.5	16.7 ± 0.6	3.5	0.1055
TL (cm) *	3.5	3.2	3.8	3.2	3.8	3	3.8	3.4	3.2	3.4 ± 0.3	28.5	0.0011
PL (mm)	9	8.5	8.6	9.3	9.3	8.9	8.9	9.2	9.5	9.0 ± 0.3	0.3	0.6025
PW (mm)	5.4	5.5	5.1	5.6	5.5	5.4	5.2	5.4	5.6	5.4 ± 0.2	4.4	0.0730
SL (mm)	6.2	5.7	6	6.5	6.7	6.2	6.3	6.5	6.8	6.3 ± 0.3	0.0	0.8859
DBN (mm)	2.9	2.9	2.8	3	3	2.8	2.7	2.9	3.1	2.9 ± 0.1	1.2	0.3015
ED ^L^ (mm)	2	1.8	1.8	1.8	1.8	1.9	1.9	1.8	1.9	1.9 ± 0.1	0.5	0.5140
ED ^R^ (mm)	2	2	1.9	1.8	1.9	2	1.9	1.8	2	1.9 ± 0.1	0.0	0.9228
BW (g)	3.7	3.5	3.3	3.8	3.5	2.7	3.2	3.3	3.3	3.4 ± 0.3	0.2	0.6404
DS	19	19	19	19	19	19	19	19	19	19.0 ± 0.0		
VS *	167	165	171	164	171	166	171	164	165	167.1 ± 2.9	28.5	0.0011
ScS *	58	49	59	50	58	49	60	50	48	53.4 ± 4.8	253.9	0.0000
SS ^L^	7	7	7	7	7	7	7	7	7	7.0 ± 0.0		
SS ^R^	7	7	7	8	7	8	7	7	7	7.2 ± 0.4	2.1	0.1930
PrS ^L^	2	1	1	1	1	1	1	1	1	1.1 ± 0.3	1.3	0.2924
PrS ^R^	1	1	1	1	1	1	1	1	2	1.1 ± 0.3	0.8	0.4071
PoS ^L^	3	3	3	3	3	2	3	3	2	2.8 ± 0.4	2.1	0.1930
PoS ^R^	3	3	3	3	3	2	3	3	3	2.9 ± 0.3	0.8	0.4071
TS ^1L^	1	1	1	1	1	1	1	1	1	1 ± 0.0		
TS ^2L^	1	1	1	1	2	2	2	2	2	1.6 ± 0.5	0.1	0.7980
TS ^1R^	1	1	1	1	1	1	1	1	1	1 ± 0.0		
TS ^2R^	2	1	1	2	1	3	1	2	2	1.7 ± 0.7	3.3	0.1118

## Data Availability

The data supporting the findings of this study are contained within the article.
